# Randomized controlled trials comparing surgery to non-operative management in neurosurgery: a systematic review

**DOI:** 10.1007/s00701-019-03849-w

**Published:** 2019-02-23

**Authors:** Enrico Martin, Ivo S. Muskens, Joeky T. Senders, Aislyn C. DiRisio, Aditya V. Karhade, Hasan A. Zaidi, Wouter A. Moojen, Wilco C. Peul, Timothy R. Smith, Marike L. D. Broekman

**Affiliations:** 1 Computational Neurosciences Outcomes Center, Department of Neurosurgery, Brigham and Women’s Hospital, Harvard Medical School, 60 Fenwood Road, Boston, MA 02115 USA; 2 Department of Plastic and Reconstructive Surgery, University Medical Center Utrecht, Utrecht, The Netherlands; 3 Department of Neurosurgery, Haaglanden Medical Center, The Hague, The Netherlands; 4 Center for Genetic Epidemiology, Department of Preventive Medicine, Keck School of Medicine, University of Southern California, Los Angeles, CA USA; 5 Icahn School of Medicine at Mount Sinai, New York, NY USA; 6 Department of Neurosurgery, Haga Teaching Hospital, The Hague, The Netherlands; 7 Department of Neurosurgery, Leiden University Medical Center, PO Box 9600, 2300 RC Leiden, The Netherlands

**Keywords:** Randomized controlled trial, Neurosurgery, Trial design, Trial registration, Systematic review, Trial quality

## Abstract

**Background:**

A randomized controlled trial (RCT) remains the pinnacle of clinical research design. However, RCTs in neurosurgery, especially those comparing surgery to non-operative treatment, are rare and their relevance and applicability have been questioned. This study set out to assess trial design and quality and identify their influence on outcomes in recent neurosurgical trials that compare surgery to non-operative treatment.

**Methods:**

From 2000 to 2017, PubMed and Embase databases and four trial registries were searched. RCTs were evaluated for study design, funding, adjustments to reported outcome measures, accrual of patients, and academic impact.

**Results:**

Eighty-two neurosurgical RCTs were identified, 40 in spine disorders, 19 neurovascular and neurotrauma, 11 functional neurosurgery, ten peripheral nerve, and two pituitary surgery. Eighty-four RCTs were registered, of which some are ongoing. Trial registration rate differed per subspecialty. Funding was mostly from non-industry institutions (58.5%), but 25.6% of RCTs did not report funding sources. 36.4% of RCTs did not report a difference between surgical and non-operative treatment, 3.7% favored non-operative management. Primary and secondary outcome measures were changed in 13.2% and 34.2% of RCTs respectively and varied by subspecialty. 41.9% of RCTs subtracted ≥ 10% of the anticipated accrual and 12.9% of RCTs added ≥ 10%. 7.3% of registered RCTs were terminated, mostly due to too slow recruitment. Subspecialty, registration, funding, masking, population size, and changing outcome measures were not significantly associated with a reported benefit of surgery. High Jadad scores (≥ 4) were negatively associated with a demonstration of surgical benefit (*P* < 0.05).

**Conclusions:**

Neurosurgical RCTs comparing surgical to non-operative treatment often find a benefit for surgical treatment. Changes to outcome measurements and anticipated accrual are common and funding sources are not always reported.

**Electronic supplementary material:**

The online version of this article (10.1007/s00701-019-03849-w) contains supplementary material, which is available to authorized users.

## Introduction

Most neurosurgical procedures are the result of continuous improvement and evolution of existing practices, and are rarely compared with non-operative management. The randomized controlled trial (RCT) is commonly regarded as the pinnacle of trial design and is thought to produce the highest quality evidence to prove effectiveness of interventions [21]. Conducting a randomized controlled trial in neurosurgery is regarded as challenging due to difficulties with patient inclusion, surgical selection bias, finding an appropriate control group, defining clinically relevant outcomes, perceived lack of equipoise, and providing a conclusive answer to its initial question [3, 22]. Most innovation in neurosurgery takes place without formalized oversight, which some justify given the unique nature of surgery, an idea referred to as “surgical exceptionalism” [15]. Perhaps as a result, RCTs in neurosurgery are conducted relatively infrequently, and their quality has been suggested to be poor [4, 12, 18]. This may be especially true for trials comparing neurosurgical procedures to non-operative management, rather than to a different neurosurgical procedure or the use of a medical device [7, 11, 22]. In many other surgical fields, including ophthalmologic surgery and vascular surgery, RCT quality seems to be poor, even though the quality of surgical RCTs seems to be improving [2, 5, 26].

Neurosurgical trial quality, registration, and reporting have been questioned as well [17, 18]. These factors may affect reported outcomes and complicate their interpretability and relevance to neurosurgical care. In this systematic review, the literature was evaluated for RCTs that compared a neurosurgical procedure with non-operative management. In addition to evaluating neurosurgical RCT design, quality, conduction, and reported outcomes, this review aims to assess what trial characteristics are associated with a reported surgical benefit.

## Methods

A systematic search was performed in both PubMed and Embase databases according to the Preferred Reporting Items for Systematic Reviews and Meta-Analysis (PRISMA) guidelines, [24] in order to identify all potentially relevant trials between 2000 and 2017. The search string was drafted with the help of a professional librarian using search terms related to “neurosurgery” together with specific neurosurgical procedures and synonyms of “randomized trial.” The databases were only searched for RCTs published after 2000 to identify relatively recent trials. The exact search syntaxes for PubMed and Embase are shown in Supplementary Table S1. Studies were included if they described data from a randomized controlled trial that compared any form of surgery to a non-surgical group. Only incisional surgery was regarded as surgical treatment, but sham surgery was regarded as non-surgical. Papers were excluded that (1) were not part of a trial of which the results were already published, (2) had no full text available, or (3) were not written in English, Dutch, German, or French. The initial review was carried out by four independent authors (EM, IM, JS, AD). Disagreements were solved by discussion in which one additional author was involved (MB). The number of published papers per trial was recorded and included published design/protocol, pilot studies, and early results. Data were extracted from the first published paper on main results. These included (a) trial start and end dates, (b) neurosurgical subspecialty, (c) countries involved, (d) number of countries involved, (e) number of participating centers, (f) funding source (non-industry, industry, or not reported), (g) total number of anticipated and included patients, (h) patients per study arm, (i) masking, and (j) if the outcome favored surgery or non-operative treatment. Scopus was consulted for the number of times the first results of the study were cited. The impact factor of the journal was determined as the journal’s indicated impact factor of 2016. Jadad scales were calculated for each trial to measure study quality [8]. The Jadad scale is the most widely used tool to assess methodological quality of a clinical trial giving scores zero (very poor) to five (rigorous) for randomization, blinding, and description of withdrawals and dropouts.

Four trial registries (ClinicalTrials.gov, EudraCT, ISRCTN, and ICTRP) were also searched with synonyms of “neurosurgery” and neurosurgical procedures. All randomized trials investigating a neurosurgical treatment to a non-surgical treatment were included. Registry data and published protocols were used to determine if and what changes, if any, were made to primary and secondary outcome measurements in protocols as compared to the first published trial results. Additionally, the anticipated accrual of patients was evaluated to determine whether it was met. The current status of registered trials was also noted.

Methodological characteristics (as listed above) were evaluated for association with benefit for either the surgical or non-surgical arm by univariate logistic regression. Statistical analyses and data visualization were conducted using R version 3.4.3 (R Core Team, Vienna, Austria, 2017).

## Results

**Fig. 1:**
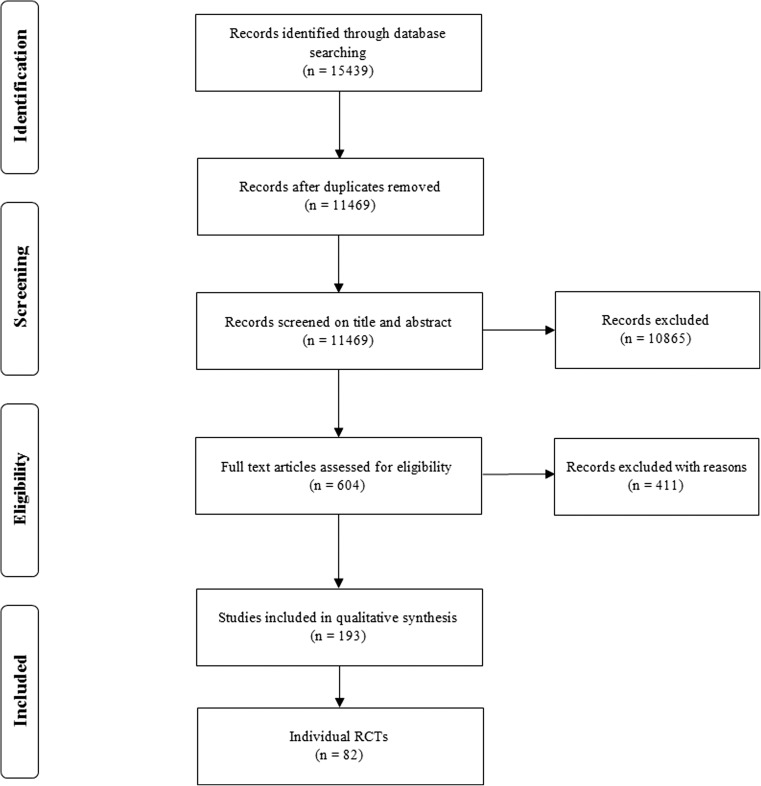
Flowchart depicting study selection

After removal of duplicates, a total of 11,469 citations were identified in PubMed and Embase databases. Six hundred four potentially relevant articles were selected through title/abstract screening, of which 193 articles were selected for qualitative synthesis after full-text screening (Fig. 1). A total of 82 individual RCTs were identified (Table 1, Supplementary Table S2). By search trial registries, a total of 84 RCTs were found.

**Table 1:** RCT demographics per subspecialty

		Total	Spinal	V & NT	Functional	PNS	Pituitary
No. trials		82	40 (48.8%)	19 (23.2%)	11 (13.4%)	10 (12.2%)	2 (2.4%)
Registered		38 (46.3%)	15 (37.5%)	13 (68.4%)	5 (45.5%)	5 (50%)	0 (0%)
No. publications	Median (IQR)	2 (1–3)	2 (1–4)	1 (1–2)	2 (1–2)	1 (1–2)	1 (1–1)
No. centers	Multicentered	48 (58.5%)	22 (55%)	16 (84.2%)	7 (63.6%)	2 (20%)	1 (50%)
	Single-centered	30 (36.6%)	15 (37.5%)	2 (10.5%)	4 (36.4%)	8 (80%)	1 (50%)
	Unknown	4 (4.9%)	3 (7.5%)	1 (5.3%)	0 (0%)	0 (0%)	0 (0%)
	Median (IQR)	3.5 (1.0–13.0)	3.0 (1.0–9.0)	18.5 (6.0–47.3)	3.0 (1.0–8.5)	1.0 (1.0–1.0)	23.5 (12.3–34.8)
No. countries	Median (IQR)	1.0 (1.0–1.0)	1.0 (1.0–1.0)	1.0 (1.0–7.8)	1.0 (1.0–1.0)	1.0 (1.0–1.0)	4.0 (2.5–5.5)
Duration (mo)	Median (IQR)	42 (27.8–68)	42 (35.5–60)	63 (21.8–90.8)	47 (39.8–58)	18 (12.5–36.5)	*NA*
No. patients	Total (median)	95 (50–175)	98 (63–178)	112 (35–300)	48 (35–118)	108 (52–119)	62 (42–81)
	Sx (median)	48 (26–87)	50 (30–87)	61 (21–175)	26 (16–39)	54 (18–60)	30.5 (21–40)
	Non-Sx (median)	47 (24–82)	49 (30–71)	73 (19–164)	21 (16–39)	54 (30–60)	31 (21–41)
Masking	Double blind	7 (8.5%)	3 (7.5%)	0 (0%)	4 (36.4%)	0 (0%)	0 (0%)
	Single blind	26 (31.7%)	8 (20%)	9 (47.4%)	5 (45.5%)	4 (40%)	0 (0%)
	Open label	49 (59.8%)	29 (72.5%)	10 (52.6%)	2 (18.2%)	6 (60%)	2 (100%)
Outcome	Surgical	52 (63.4%)	23 (57.5%)	13 (68.4%)	8 (72.7%)	8 (80%)	0 (0%)
	Non-operative	3 (3.7%)	2 (5%)	1 (5.3%)	0 (0%)	0 (0%)	0 (0%)
	No difference	27 (32.9%)	15 (37.5%)	5 (26.3%)	3 (27.3%)	2 (20%)	2 (100%)
Funding	Non-industry	48 (58.5%)	25 (62.5%)	11 (57.9%)	7 (63.6%)	5 (50%)	0 (0%)
	Industry	13 (15.9%)	7 (17.5%)	1 (5.3%)	4 (36.4%)	0 (0%)	1 (50%)
	Not reported	21 (25.6%)	8 (20%)	7 (36.8%)	0 (0%)	5 (50%)	1 (50%)
No. citations	Median (IQR)	95 (21.8–296.0)	127.5 (22.8–286.0)	135 (30.5–331.0)	258 (64.5–1058.0)	48 (3.3–86.5)	40 (26.0–54.0)
Impact factor	Median (IQR)	6.1(2.4–39.3)	3.4 (2.1–32. 1)	23.5 (3.6–44.0)	23.5 (8.9–48.6)	8.2 (3.0–15.0)	3.5 (3.5–3.5)
Jadad	Median (IQR)	3 (2–3)	2.5 (2–3)	3 (2–3)	3 (2–4)	3 (1.25–3)	1 (1–1)

*IQR* interquartile range, *mo* months, *No.* number of, *PNS* peripheral nerve surgery, *SD* standard deviation, *Sx* surgical arm, *V & NT* neurovascular and neurotrauma

### Study characteristics

Of all included randomized trials, 40 (48.8%) could be categorized as spine, 19 (23.2%) neurovascular and neurotrauma, 11 (13.4%) functional-, 10 (12.2%) peripheral nerve–, and 2 (2.4%) pituitary-surgery (Table 1). Overall, a median of two papers (IQR 1–3) were published per trial, with spinal (2, IQR 1–4) and functional (2, IQR 1–2) subspecialties having most publications per RCT. Trial registration was relatively the highest in vascular neurosurgery and neurotrauma (68.4%) and lowest in spine surgery (37.5%). Twenty RCTs were multicentre trials, but this was only the case in 20% of peripheral nerve surgery trials (*n* = 2). Median time to trial inclusion completion was 42 months (IQR 27.8–68.0). RCTs in peripheral nerve surgery had the lowest median time to study completion (18 months, IQR 12.5–36.5). Overall, the median number of patients included in an RCT was 95 (IQR 50–175), with relatively smaller populations in functional neurosurgery trials (48, IQR 35–118). Study arms were generally distributed evenly (Table 1). Overall, most trials were open-label (59.8%) and double-blind trials were relatively rare (8.5%). Double-blind trials were most common in functional neurosurgery (36.4%). Funding was usually from non-industry parties (58.5%). However, the funding was not reported in 25.6% of RCTs. Median Jadad scores were 3 (IQR 2–3).

### Factors associated with trial outcome

The majority of trials reported a favorable outcome for surgical intervention (63.4%) (Table 1). Only 3.7% of all trials reported a beneficial effect of the non-surgical intervention, while the rest (32.9%) did not find any statistical differences. High Jadad scores (≥ 4) were negatively associated with the demonstration of a surgical benefit (OR 0.10, 95% CI 0.01–0.89). None of the other trial characteristics showed a significant relationship to surgical benefit (all *P* values > 0.05, Table 2).

**Table 2:** Univariate analysis of trial outcome

		No surgical benefit (*N* = 30)	Surgical benefit (*N* = 52)	OR (95%-CI)	*P* value
Subspecialty (%)	Spinal	17 (56.7)	23 (44.2)	Ref.	
	Vascular	6 (20.0)	13 (25.0)	1.60 (0.52–5.35)	0.42
	Functional	3 (10.0)	8 (15.4)	1.97 (0.49–10.0)	0.36
	PNS	2 (6.7)	8 (15.4)	2.96 (0.64–21.3)	0.20
	Pituitary	2 (6.7)	0 (0.0)	NA	
Registered (%)	Not registered	16 (53.3)	28 (53.8)	Ref.	
	Registered	14 (46.7)	24 (46.2)	0.98 (0.40–2.43)	0.96
Funding (%)	Non-industry	20 (66.7)	28 (53.8)	Ref.	
	Industry	5 (16.7)	8 (15.4)	1.14 (0.33–4.26)	0.84
	Unknown	5 (16.7)	16 (30.8)	2.29 (0.75–7.92)	0.16
Multicenter (%)	Singlecentre	9 (30.0)	21 (40.4)	Ref.	
	Multicentre	20 (66.7)	28 (53.8)	0.60 (0.23–1.58)	0.30
	NA	1 (3.3)	3 (5.8)	1.29 (0.12–14.09)	0.84
Masking (%)	Open label	16 (53.3)	33 (63.5)	Ref.	
	Single blind	9 (30.0)	17 (32.7)	0.92 (0.34–2.56)	0.86
	Double blind	5 (16.7)	2 (3.8)	0.19 (0.03–1.01)	0.07
Number of patients (%)	< 100	13 (43.3)	29 (55.8)	Ref.	
	≥ 100	17 (56.7)	23 (44.2)	0.61 (0.24–1.49)	0.28
Change in primary outcome measure (%)	No change	14 (46.7)	19 (36.5)	Ref.	
	Change	1 (3.3)	4 (7.7)	2.95 (0.38–61.1)	0.36
	Unknown	15 (50.0)	29 (55.8)	1.42 (0.56–3.64)	0.46
Change in secondary outcome measure (%)	No change	8 (26.7)	14 (26.9)	Ref.	
	Change	5 (16.7)	9 (17.3)	1.03 (0.26–4.34)	0.97
	Unknown	17 (56.7)	29 (55.8)	0.97 (0.33–2.77)	0.96
Jadad (%)	Jadad < 3	11 (36.7)	27 (51.9)	Ref.	
	Jadad ≥ 3	19 (63.3)	25 (48.1)	0.54 (0.21–1.33)	0.18
	Jadad < 4	25 (83.3)	51 (98.1)	Ref.	
	Jadad ≥ 4	5 (16.7)	1 (1.9)	0.10 (0.01–0.89)	0.01

### Changes in primary and secondary outcome measures

**Fig. 2:**
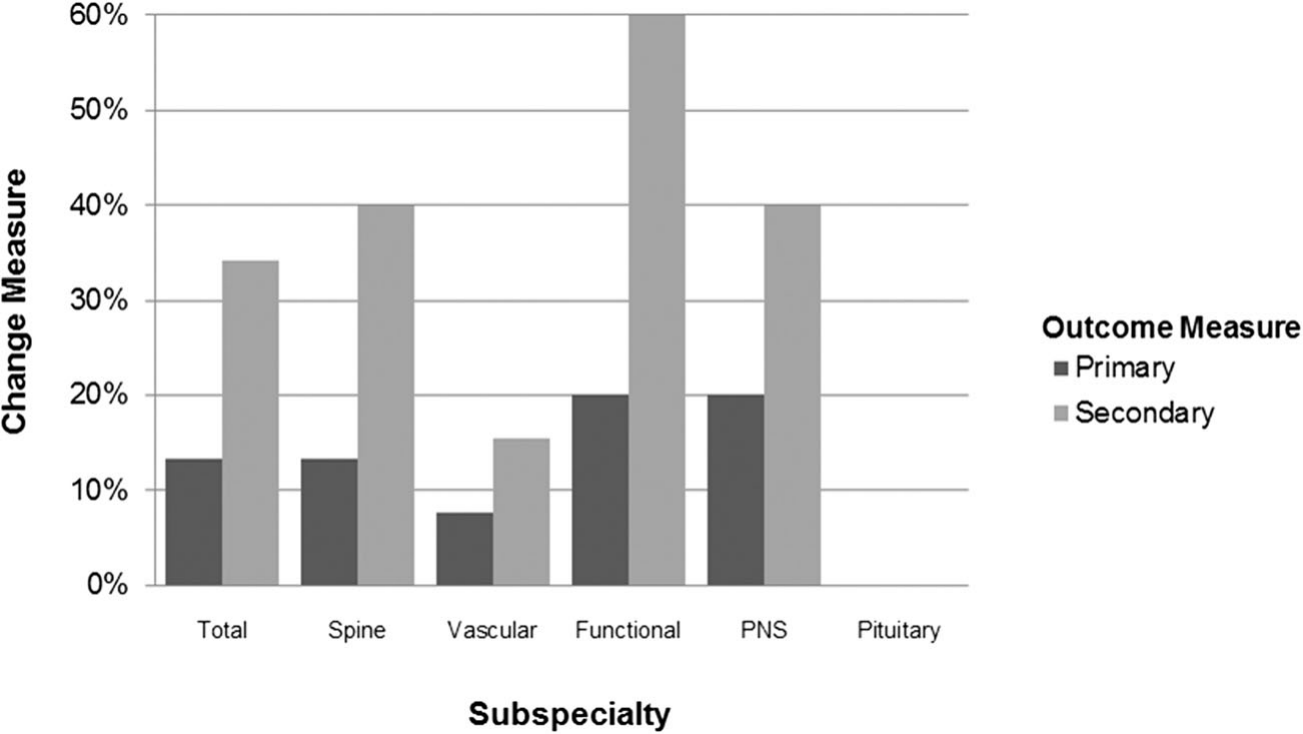
Changes in outcome measures per subspecialty

Only registered trials (*n* = 38) were available for assessment of changes in primary and secondary outcomes. 13.2% of these RCTs changed their primary outcome measurement between registration and publication (*n* = 5, Fig. 2). 60% of these changes were simple changes to the primary outcome measure (n = 3), 20% added a primary outcome measure (*n* = 1), and 20% removed one of the primary outcome measures (*n* = 1, Table 3). Secondary outcome measures were changed in 34.2% of all RCTs (*n* = 16). 50% were simply changed (*n* = 8), 37.5% had an additional secondary outcome measure (*n* = 6), and 12.5% of studies removed one or more of their secondary outcome measures (*n* = 2).

**Table 3:** Changes in primary and secondary outcome measures

		Percentage (%)
Change primary outcome	Changed	60
	Added	20
	Removed	20
Change secondary outcome	Changed	50
	Added	37.5
	Removed	12.5

### Trial continuation and anticipated accrual of patients

65.9% of registered RCTs were completed and 26.8% was still ongoing (Table 4). 7.3% of RCTs had been terminated. This was most commonly due to slow recruitment or meeting a pre-specified futility boundary. The initial anticipated accrual was lowered by more than 10% in 41.9% of all RCTs. The accrual was diminished by 58.5% on average (SD 25.1%). In 12.9% of trials, initial estimated accrual surpassed 110% of planned patient enrolment (mean added percentage 41.2, SD 36.0%).

**Table 4:** Trial registration data

		Percentage
RCT status	Completed	65.9%
	Active	26.8%
	Terminated	7.3%
Accrual patients	Subtracted > 10%	41.9%
	Mean (SD)	58.5% (25.1)
	Added > 10%	12.9%
	Mean (SD)	41.2% (36.0)

### Academic impact

The median number of citations per study was 95 (IQR 21.8–296.0, Table 1). Peripheral nerve surgery and pituitary trials had the lowest median number of citations (48, IQR 3.3–86.5 and 40, IQR 26.0–54.0, respectively). Median impact factor of the journal in which the study was published was 6.1 (IQR 2.4–39.3). Functional neurosurgery trials had the highest median impact factor at 23.5 (IQR 8.9–48.6). The median number of citations and impact factor did not differ for trial outcome overall (*P* = 0.33 and *P* = 0.73, respectively, Table 5). Post-hoc analyses also did not reveal any significant difference in number of citations or impact factor between trial outcomes (all *P* > 0.05).

**Table 5:** Average academic impact per outcome

	Citations median (IQR)	*P* value	Impact factor median (IQR)	*P* value
Surgical	103.0 (19.5–331.5)	0.33	6.1 (2.3–44.0)	0.73
Non-surgical	119.0 (0–NA)		2.1 (2.1–NA)	
No difference	72.0 (24.0–301.0)		5.8 (2.4–26.5)	

## Discussion

The aim of this study was to evaluate trial outcomes in recent neurosurgical RCTs comparing surgery to non-operative treatment. Most studies found superior outcomes for surgery, while non-operative treatment rarely resulted in superior outcomes. The considerable academic impact of the studies indicates that the results of neurosurgical RCTs seem to be of value to the neurosurgical community. However, their clinical impact remains a challenge to determine and it is uncertain to what extent neurosurgical practice was changed as a result of the results of neurosurgical RCTs. It has been suggested that the absence of a surgical benefit promotes non-operative management.

The authors of the identified RCTs are to be applauded for their considerable continuous efforts, given that many trials were registered and had published their protocol. However, this study identified several challenges common among neurosurgical RCTs. The overall quality of the identified studies based on the Jadad score could be considered poor. Also, funding sources were not reported consistently among all studies identified and many trials were not registered. Changes to primary or secondary outcome measures occurred frequently but were not shown to influence whether surgery was found to be superior to a non-operative treatment.

### Trial registration and outcome measurement

Results of previous studies have suggested that differences between registered and published outcomes are common among RCTs in general surgery and that these differences are not related to funding sources [10, 23]. This is in line with the results of this study. Interestingly, it has been shown that 91.7% of surgical trials that changed outcome measures published significant results [13]. This is similar to findings in cardiology, rheumatology, and gastroenterology [20]. Furthermore, a recent study of RCTs in spine surgery showed that statistical findings could be considered weak as the addition of only few events or non-events would have changed the significance of the reported finding [4].

### Trial quality

This study found a generally poor quality of RCTs based on Jadad scores. These results are in line with two previous studies of neurosurgical RCTs [12, 18]. The study by Mansouri et al. also identified that trials that evaluated surgical procedures met their target inclusion less often than trials that evaluated drugs or medical devices [18]. This may implicate that conducting a trial for surgical procedures is more difficult but may also be the result of bias. Kiehna et al. showed that studies published in high-impact journals had higher mean CONSORT and Jadad scores [12]. Importantly, superiority of the surgical approach did not affect academic impact. It should, however, be noted that both the CONSORT and Jadad scores have limits and do not incorporate all potential (methodological) challenges and limitations of RCTs, especially of surgical RCTs.

### Strengths and limitations

This is the first study that sought to evaluate which trial characteristics were associated with the identification of a surgical superiority compared to non-operative treatment in neurosurgical RCTs. Both MEDLINE search engines and trial registries were extensively evaluated. The findings provide a valuable insight into the frequency of trial cessation, adjustment of trial design, and quality of reporting, which may provide useful insights for future neurosurgical RCTs.

There are also several limitations to this study. The search engines and registries only provided a relatively small number of RCTs. There is a possibility that not registered or unpublished trials were not identified. This may have caused selection bias influencing the findings in this analysis of studies. Selection bias by reviewers and publication bias may have occurred for studies that did not find statistically significant results, or an outcome favoring surgery. What’s more, most trials were conducted by surgeons, which may have given inherent bias to preferred outcomes. This may explain why only a very low number of studies were identified that found a neurosurgical procedure to be associated with inferior outcomes. Only RCTs published after 2000 were included, which further limits the number of trials included. Analysis to determine which trial characteristics may be associated with a surgical benefit was complicated because only a minority of the published trials had also been registered and had their protocol available. Therefore, it was not possible to evaluate whether protocols were changed for unregistered studies, which may have provided additional valuable insights. This study is also limited by the sole inclusion of RCTs that compared a surgical procedure with non-operative management. This mainly has implications for oncologic RCTs, as often different radiation and medical regimens are compared instead of a surgical procedure [17]. Moreover, although the Jadad score is the most commonly used assessment tool for trial quality, it does not take allocation concealment into account. This may potentially bias results. Lastly, non-quantifiable trial characteristics that were not compared in this study may influence these findings.

Future studies on neurosurgical RCTs could study subspecialty specific trial characteristics even more profoundly and their influence on trial quality and findings. Also, investigating trials comparing a novel neurosurgical procedure to current standard of practice in a similar fashion to this study may give insightful information on how to better interpret their results. Finally, evaluation of neurosurgical RCTs could be aided by the introduction of a trial registry that is specific to neurosurgery and takes into account the unique challenges of a neurosurgical RCT.

### Implication for future neurosurgical RCTs

The findings of this study regarding trial registration, patient accrual, trial completion, publication, and alteration of outcome measures provide suggestions for improvement of future neurosurgical RCTs. Neurosurgical RCTs should seek to answer questions that live among the neurosurgical community and can be answered by an RCT. This requires true equipoise, the availability of patients, and sufficient funding among other things. Other trial designs, such as a prospective observational study, should be considered if they are more suitable to answer unresolved controversies in neurosurgery [16].

Most journals nowadays require an RCT to be registered, disclose their funding sources, and publish a protocol to increase transparency. The protocol should ideally be published in a neurosurgical journal to provide a neurosurgical readership the possibility to suggest alterations to the trial design to improve trial quality and make the potential findings as relevant as possible. Alterations to outcome measures should always be disclosed to readers together with a reason for this alteration. Investigators should be realistic about inclusion and exclusion criteria to meet the estimated number of patients to be included and should optimize the inclusion process. Similar to our findings, another study found trial discontinuation to be common in neurosurgical trials in general, most commonly due to slow recruitment [9]. A pilot study to evaluate the patient inclusion process that also provides an estimate of the outcome measure may prevent inadequate recruitment [14]. Others found that telephone reminders to non-responders, opt-out procedures, and financial incentives may help patient inclusion [25].

Although conducting a neurosurgical RCT may be considered burdensome, they should, in the end, provide answers of the highest possible quality that are relevant to the neurosurgical community. A well-designed and conducted trial could make sure that the effort and funding put in do not go to waste. A trial registry specific to neurosurgery might help address some of the issues affecting the quality of RCTs in neurosurgery. Alternatively, comparative effectiveness research (CER) or pragmatic RCTs may also provide valuable insights and have been suggested to be of great use in spine surgery [6, 19]. Furthermore, “big data” may prove an important tool for identification of trial-worthy innovations. The digitization of medical records, introduction of patient outcome measures, and increasing computational capacity have resulted in the availability of the most comprehensive pre-trial data yet, despite varying quality. These data sets could become of high value by itself in cases where RCTs are not feasible [1].

## Conclusion

RCTs comparing surgical to non-operative treatment are rare in neurosurgery and the majority identify a benefit for surgical treatment. The quality of RCTs is generally low and outcome measurements frequently change. Trial registration is done in half of all RCTs and funding sources are not always reported. Furthermore, the anticipated accrual of patient was often greater than the number of included patients. Success of future neurosurgical RCTs could be improved by trial and protocol registration prior to patient inclusion, pilot studies, and use of big data.

## Electronic supplementary material

Table S1 Search strategy. This table shows the search strategy for PubMed and Embase databases (DOCX 24 kb)

Table S2 All 84 individual randomized controlled trials (DOCX 37 kb)
