# Supplementary material for: Randomized controlled trials comparing surgery to non-operative management in neurosurgery: a systematic review
Source: Acta Neurochir (Wien). 2019 Feb 23;161(4):627–34. doi: 10.1007/s00701-019-03849-w (PMC6431301; doi:10.1007/s00701-019-03849-w)
Supplement: Supplementary file 1 — Search strategy. This table shows the search strategy for PubMed and Embase databases (DOCX 24 kb) [file 701_2019_3849_MOESM1_ESM.docx]

**Supplementary Table S1**

| **PubMed search:**  **1-2017** | ((((((((neurosurg*[tw] OR cranial surg*[tw] OR spine surg*[tw] OR spinal surg*[tw] OR Temporal Lobectom*[tw] OR cortectom*[tw] OR "Brain Tissue Transplantation"[tw] OR "Cerebral Decortication"[tw] OR Hemispherectom*[tw] OR Cerebrospinal Fluid Shunt*[tw] OR Ventriculoperitoneal Shunt*[tw] OR CSF Shunt*[tw] OR VP Shunt*[tw] OR "Trephining"[tw] OR "Parasympathectomy"[tw] OR "Sympathectomy"[tw] OR neurectom*[tw] OR spinal fusion[tw] OR disc fusion[tw] OR ACDF[tw] OR spinal decompression[tw] OR "Microvascular Decompression Surgery"[tw] OR "Nerve Transfer"[tw] OR "Split-Brain Procedure"[tw] OR "Neuronavigation"[tw] OR "Radiosurgery"[tw] OR (SRS[tw] NOT sexual[tw]) OR gamma knife[tw] OR cyber knife[tw] OR stereotactic radiotherapy[tw] OR deep brain stimulation[tw] OR (DBS[tw] NOT double bare metal stent[tw]) OR neurostimulation[tw] OR neurostimulator[tw] OR spinal cord stimulator*[tw] OR spinal cord stimulation[tw] OR "Diskectomy"[tw] OR disc replacement[tw] OR spinal decompression[tw] OR spinal cord decompression[tw] OR Ventriculostom*[tw] OR Craniotom*[tw] OR cranioplast*[tw] OR Decompressive Craniectom*[tw] OR Corpus callosotomy[tw] OR Vagotom*[tw] OR Ganglionectom*[tw] OR Axotom*[tw] OR Cordotom*[tw] OR Ganglionectom*[tw] OR Rhizotom*[tw] OR Vagotom*[tw] OR Foraminotom*[tw] OR Hypophysectom*[tw] OR Laminectom*[tw] OR laminotom*[tw] OR Laminoplast*[tw] OR Neuroendoscop*[tw] OR endonasal[tw] OR Pallidotom*[tw] OR thalamotom*[tw] OR cortical resection*[tw] OR Psychosurg*[tw] OR microvascular decompression surgery[tw] OR Radiosurg*[tw] OR Diskectom*[tw] OR Discectom*[tw] OR cranial vault resconstruction[tw] OR cranial vault remodeling[tw] OR craniosynostosis surgery[tw] OR auditory brainstem implant[tw] OR transphenoidal surger*[tw] OR cerebral stent[tw] OR cerebral stents[tw] OR cerebral stenting[tw] OR carotid stent[tw] OR carotid stents[tw] OR carotid stenting[tw] OR carotid endarterectom*[tw] OR (CEA[tw] NOT carcinoembryonic antigen[tw]) OR (angioplasty[tw] NOT (cardiac[tw] OR coronary[tw])) OR aneurysm coiling[tw] OR aneurysm clipping[tw] OR neurosurgical clipping[tw] OR endovascular coiling[tw] OR cerebral bypass[tw] OR cranial bypass[tw] OR middle cerebral artery bypass[tw] OR ((embolization[tw] OR resection[tw]) AND (AVM[tw] OR arteriovenous malformation[tw])) OR ((resection[tw] OR resect[tw] OR debulk*[tw]) AND (brain[tw] OR cranial[tw] OR intracranial[tw] OR cerebral[tw] OR spinal[tw] OR CNS[tw] OR glioma[tw] OR glioblastoma[tw] OR meningioma[tw] OR astrocytoma[tw] OR GBM[tw] OR neuroma[tw] OR pituitary tumor*[tw] OR lymphoma[tw] OR brain tumor[tw] OR brain metastasis[tw] OR brain metastases[tw])) OR "Neurosurgery"[Mesh] OR "Neurosurgical Procedures"[Mesh] OR "Spine/surgery"[mesh] OR "Spinal Diseases/surgery"[mesh] OR "Brain/surgery"[mesh] OR "Brain Diseases/surgery"[mesh] OR "Central Nervous System/surgery"[mesh] OR "Central Nervous System Diseases/surgery"[mesh] OR "Nervous System/surgery"[mesh] OR "Nervous System Diseases/surgery"[mesh]))) AND ((randomized controlled trial[tw] OR randomized controlled study[tw] OR randomly assigned[tw] OR randomized trial[tw] OR randomized, double-blind, placebo-controlled trial[tw] OR randomized, double blind, controlled trial[tw] OR randomized, double blind[tw] OR randomized trial[tw] OR prospective, double blind[tw] OR controlled clinical trial[tw] OR randomized clinical trial[tw] OR double blind[tw] OR prospective clinical trial[tw] OR randomised controlled trial[tw] OR randomised controlled study[tw] OR randomised trial[tw] OR randomised, double-blind, placebo-controlled trial[tw] OR randomised, double blind, controlled trial[tw] OR randomised, double blind[tw] OR randomised trial[tw] OR randomised clinical trial[tw] OR ("Randomized controlled trial"[publication type])))) |
| --- | --- |
| **Embase search:**  **1-2017** | (neurosurg* or cranial surg* or spine surg* or spinal surg* or Temporal Lobectom* or cortectom* or "Brain Tissue Transplantation" or "Cerebral Decortication" or Hemispherectom* or Cerebrospinal Fluid Shunt* or Ventriculoperitoneal Shunt* or CSF Shunt* or VP Shunt* or "Trephining" or "Parasympathectomy" or "Sympathectomy" or neurectom* or spinal fusion or disc fusion or ACDF or spinal decompression or "Microvascular Decompression Surgery" or "Nerve Transfer" or "Split-Brain Procedure" or "Neuronavigation" or "Radiosurgery" or (SRS not sexual) or gamma knife or cyber knife or stereotactic radiotherapy or deep brain stimulation or (DBS not double bare metal stent) or neurostimulation or neurostimulator or spinal cord stimulator* or spinal cord stimulation or "Diskectomy" or disc replacement or spinal decompression or spinal cord decompression or Ventriculostom* or Craniotom* or cranioplast* or Decompressive Craniectom* or Corpus callosotomy or Vagotom* or Ganglionectom* or Axotom* or Cordotom* or Ganglionectom* or Rhizotom* or Vagotom* or Foraminotom* or Hypophysectom* or Laminectom* or laminotom* or Laminoplast* or Neuroendoscop* or endonasal or Pallidotom* or thalamotom* or cortical resection* or Psychosurg* or microvascular decompression surgery or Radiosurg* or Diskectom* or Discectom* or cranial vault resconstruction or cranial vault remodeling or craniosynostosis surgery or auditory brainstem implant or transphenoidal surger* or cerebral stent or cerebral stents or cerebral stenting or carotid stent or carotid stents or carotid stenting or carotid endarterectom* or (CEA not carcinoembryonic antigen) or (angioplasty not (cardiac or coronary)) or aneurysm coiling or aneurysm clipping or neurosurgical clipping or endovascular coiling or cerebral bypass or cranial bypass or middle cerebral artery bypass or ((embolization or resection) and (AVM or arteriovenous malformation)) or ((resection or resect or debulk*) and (brain or cranial or intracranial or cerebral or spinal or CNS or glioma or glioblastoma or meningioma or astrocytoma or GBM or neuroma or pituitary tumor* or lymphoma or brain tumor or brain metastasis or brain metastases))).tw. or (neurosurgery/ or auditory brain stem implantation/ or neuroendoscopy/ or neuronavigation/ or exp skull surgery/ or exp spinal cord surgery/ or exp sympathectomy/ or exp vagotomy/ or exp ventriculostomy/ or exp spine/su or exp spine disease/su or exp brain/su or exp brain disease/su or exp central nervous system/su or exp central nervous system disease/su or neurologic disease/su) AND ((randomized controlled trial or randomized controlled study or randomized trial or randomized, double-blind, placebo-controlled trial or randomized, double blind, controlled trial or randomized, double blind or randomized trial or randomized clinical trial or double blind or randomly assigned or prospective, double blind or controlled clinical trial or prospective clinical trial or randomised controlled trial or randomised controlled study or randomised trial or randomised, double-blind, placebo-controlled trial or randomised, double blind, controlled trial or randomised, double blind or randomised trial or randomised clinical trial).tw) |
