# Supplementary material for: Randomized controlled trials comparing surgery to non-operative management in neurosurgery: a systematic review
Source: Acta Neurochir (Wien). 2019 Feb 23;161(4):627–34. doi: 10.1007/s00701-019-03849-w (PMC6431301; doi:10.1007/s00701-019-03849-w)
Supplement: Supplementary file 2 — All 84 individual randomized controlled trials (DOCX 37 kb) [file 701_2019_3849_MOESM2_ESM.docx]

| **Author** | **Year** | **Indication (trial name)** | **N** | **Funding** | **Outcome study** | **Jadad** |
| --- | --- | --- | --- | --- | --- | --- |
| *Spinal disorders* | | | | | | |
| Ekman et al. | 2000 | Isthmic spondylolisthesis | 111 | Non-industry | Surgical | 3 |
| Persson et al. | 2000 | Chronic radicular neck pain | 81 | Non-industry | Surgical | 1 |
| Fritzell et al. | 2001 | Chronic lower back pain | 284 | Industry | Surgical | 3 |
| Kadanka et al. | 2002 | Cervical myelopathy | 68 | Non-industry | *NS* | 2 |
| Brox et al. | 2003 | Chronic back pain after previous operated herniated disc | 64 | Non-industry | *NS* | 3 |
| Wood et al. | 2003 | Thoracolumbar burst fracture without neurological deficit | 53 | Non-industry | *NS* | 2 |
| Buttermann et al. | 2004 | Lumbar herniated disc | 170 | *NA* | Surgical | 2 |
| Zucherman et al. | 2004 | Neurogenic claudication (X-STOP) | 200 | Industry | Surgical | 3 |
| Patchell et al. | 2005 | Metastatic epidural spinal cord compression | 101 | Non-industry | Surgical | 2 |
| Fairbank et al. | 2005 | Chronic lower back pain | 349 | Industry | *NS* | 2 |
| Osterman et al. | 2006 | Lumbar herniated disc | 56 | Non-industry | *NS* | 3 |
| Siebenga et al. | 2006 | Non-osteoporotic thoracic and lumbar fracture | 34 | Non-industry | Surgical | 2 |
| Anderson et al. | 2006 | Lumbar degenerative spondylolisthesis (X-STOP) | 75 | *NA* | Surgical | 2 |
| Weinstein et al. | 2006 | Lumbar herniated disc (SPORT) | 501 | Non-industry | *NS* | 2 |
| Weinstein et al. | 2007 | Lumbar degenerative spondylolisthesis (SPORT) | 304 | Non-industry | Surgical | 2 |
| Slatis et al. | 2007 | Lumbar spinal stenosis | 94 | Non-industry | Surgical | 3 |
| Weinstein et al. | 2008 | Lumbar spinal stenosis (SPORT) | 289 | Non-industry | Surgical | 2 |
| El Barzouhi et al. | 2008 | Sciatica | 283 | Non-industry | Surgical | 3 |
| Rousing et al. | 2009 | Osteoporotic vertebral fracture | 50 | Non-industry | Non-surgical | 3 |
| Buchbinder et al. | 2009 | Osteoporotic vertebral fracture | 78 | Non-industry | *NS* | 5 |
| Boonen et al. | 2009 | Vertebral compression fracture (FREE) | 300 | Industry | Surgical | 3 |
| Klazen et al. | 2010 | Osteoporotic vertebral fracture (Vertos II) | 202 | Non-industry | Surgical | 3 |
| Aronsohn et al. | 2010 | Sciatica | 50 | *NA* | Surgical | 1 |
| McMorland et al. | 2010 | Sciatica | 40 | Non-industry | *NS* | 2 |
| Hellum et al. | 2011 | Sciatica | 116 | *NA* | *NS* | 3 |
| Ohtori et al. | 2011 | Lumbar herniated disc | 41 | Non-industry | Surgical | 3 |
| Berenson et al. | 2011 | Vertebral compression fracture (CAFÉ) | 134 | Industry | *NS* | 3 |
| Erginousakis et al. | 2011 | Intervertebral disk herniation | 62 | *NA* | Surgical | 0 |
| Piazolla et al. | 2011 | Non-osteoporotic thoracic and lumbar fracture | 50 | Non-industry | Surgical | 2 |
| Farrokhi et al. | 2011 | Osteoporotic vertebral compression fracture | 82 | Industry | Surgical | 3 |
| Chitragran et al. | 2012 | Radicular or axial low back pain | 64 | *NA* | Surgical | 2 |
| Peris et al. | 2012 | Vertebral fractures | 125 | Non-industry | *NS* | 3 |
| Kallmes et al. | 2012 | Vertebroplasty spine fractures | 131 | Non-industry | *NS* | 5 |
| Elsawaf et al. | 2013 | Cervical tuberculosis | 29 | *NA* | Surgical | 1 |
| Engquist et al. | 2013 | Cervical radiculopathy | 63 | Non-industry | *NS* | 3 |
| Wardlaw et al. | 2013 | Soft disc herniation | 100 | Non-industry | *NS* | 2 |
| Chen et al. | 2014 | Osteoporotic vertebral fracture | 96 | *NA* | Surgical | 2 |
| Delitto et al. | 2015 | Lumbar spinal stenosis | 169 | Non-industry | *NS* | 3 |
| Clark et al. | 2016 | Osteoporotic vertebral fracture (VAPOUR) | 120 | Industry | Surgical | 5 |
| Yang et al. | 2016 | Osteoporotic vertebral fracture | 207 | Non-industry | Non-surgical | 2 |
| *Neurovascular and neurotrauma* | | | | | | |
| Mendelow et al. | 2005 | Supratentorial intracerebral hematomas (STICH) | 1033 | Non-industry | *NS* | 3 |
| Ballotta et al. | 2005 | Isolated internal carotid artery elongation | 182 | *NA* | Surgical | 3 |
| Vahedi et al. | 2007 | Middle cerebral artery infarction (DECIMAL) | 38 | Non-industry | Surgical | 2 |
| Jüttler et al. | 2007 | Middle cerebral artery infarction (DESTINY) | 32 | *NA* | *NS* | 3 |
| Halliday et al. | 2008 | Asymptomatic carotid artery stenosis (ACST) | 3120 | Non-industry | Surgical | 3 |
| Hofmeijer et al. | 2009 | Middle cerebral artery infarction (HAMLET) | 64 | Non-industry | Surgical | 3 |
| Wang et al. | 2009 | Intracerebral hemorrhage | 377 | *NA* | Surgical | 3 |
| Cooper et al. | 2011 | Diffuse traumatic brain injury (DECRA) | 155 | Non-industry | Surgical | 2 |
| Powers et al. | 2011 | Carotid occlusion (COSS) | 195 | Non-industry | *NS* | 3 |
| Hooshang et al. | 2012 | Severe head injury | 20 | *NA* | *NS* | 2 |
| Zhao et al. | 2012 | Middle cerebral artery infarction | 47 | *NA* | Surgical | 2 |
| Mould et al. | 2013 | Supratentorial intracerebral hematomas (MISTIE) | 20 | Industry | Surgical | 2 |
| Mendelow et al. | 2013 | Supratentorial intracerebral hematomas (STICH II) | 601 | Non-industry | *NS* | 3 |
| Wu et al. | 2013 | Thalamic hematoma | 30 | *NA* | Surgical | 1 |
| Jüttler et al. | 2014 | Middle cerebral arter infarction (DESTINY II) | 112 | Non-industry | Surgical | 3 |
| Miyamoto et al. | 2014 | Hemorrhagic moyamoya disease | 70 | Non-industry | Surgical | 3 |
| Rutledge et al. | 2014 | Unruptured brain arteriovenous malformation (ARUBA) | 223 | Non-industry | Non-surgical | 3 |
| Hutchinson et al. | 2016 | Traumatic intracranial hypertension (RESCUEicp) | 408 | Non-industry | Surgical | 3 |
| Wu et al. | 2017 | Basal ganglia | 27 | *NA* | Surgical | 2 |
| *Functional* | | | | | | |
| Wiebe et al. | 2001 | Temporal lobe epilepsy | 80 | Non-industry | Surgical | 3 |
| Olanow et al. | 2003 | Parkinson’s disease | 34 | Non-industry | *NS* | 5 |
| Vitek et al. | 2003 | Parkinson | 36 | Non-industry | Surgical | 2 |
| Gordon et al. | 2004 | Parkinson’s disease | 48 | Non-industry | Surgical | 3 |
| Deuschl et al. | 2006 | Parkinson’s disease | 156 | Non-industry | Surgical | 1 |
| Silverberg et al. | 2008 | Alzheimer’s disease | 215 | Industry | *NS* | 5 |
| Weaver et al. | 2009 | Parkinson | 255 | Industry | Surgical | 2 |
| Gross et al. | 2011 | Parkinson (STEPS) | 71 | Industry | *NS* | 5 |
| Kahn et al. | 2011 | Parkinson | 30 | Industry | Surgical | 1 |
| Engel et al. | 2012 | Epilepsy (ERSET) | 38 | Non-industry | Surgical | 3 |
| Thomason et al. | 2013 | Bilateral spastic cerebral palsy (SEMLS) | 19 | Non-industry | Surgical | 2 |
| *Peripheral nerve* | | | | | | |
| Gerritsen et al. | 2002 | Carpal tunnel syndrome | 176 | Non-industry | Surgical | 3 |
| Andreu et al. | 2005 | Carpal tunnel syndrome | 163 | *NA* | Surgical | 3 |
| Robert et al. | 2005 | Pudendal neuralgia | 32 | *NA* | Surgical | 2 |
| Hui et al. | 2005 | Carpal tunnel syndrome | 50 | *NA* | Surgical | 3 |
| Ucan et al. | 2006 | Carpal tunnel syndrome | 57 | *NA* | Surgical | 1 |
| Jarvik et al. | 2009 | Carpal tunnel syndrome | 116 | Non-industry | Surgical | 3 |
| Hahn et al. | 2011 | Ilioinguinal nerve entrapment | 19 | Non-industry | Surgical | 1 |
| Saboor et al. | 2015 | Carpal tunnel syndrome | 116 | *NA* | *NS* | 1 |
| Fernandes-de-las-Peñas et al. | 2015 | Carpal tunnel syndrome | 120 | Non-industry | *NS* | 3 |
| Fernandes-de-las-Peñas et al. | 2017 | Carpal tunnel syndrome | 100 | Non-industry | Surgical | 3 |
| *Pituitary* | | | | | | |
| Calao et al. | 2009 | Acromegaly | 101 | Industry | *NS* | 1 |
| Karaca et al. | 2011 | Acromegaly | 22 | *NA* | *NS* | 1 |

N: number of patients, NA: not available, NS: no difference
